# Immunotherapy for Pemphigus: Present and Future

**DOI:** 10.3389/fmed.2022.901239

**Published:** 2022-06-15

**Authors:** Huijie Yuan, Meng Pan, Hongxiang Chen, Xuming Mao

**Affiliations:** ^1^Department of Dermatology, Huazhong University of Science and Technology Union Shenzhen Hospital, Shenzhen, China; ^2^Department of Dermatology, Rui Jin Hospital, Shanghai Jiao Tong University School of Medicine, Shanghai, China; ^3^Department of Dermatology, Union Hospital, Tongji Medical College, Huazhong University of Science and Technology, Wuhan, China; ^4^Department of Dermatology, University of Pennsylvania, Philadelphia, PA, United States

**Keywords:** pemphigus, improved efficacy, immunotarget, novel therapy, clinical trial

## Abstract

Pemphigus is a chronic and severe autoimmune bullous disease caused by autoantibodies targeting adhesion molecules between keratinocytes. It requires 2–3 years on average to manage the disease. To date, although Rituximab combined with short-term systemic glucocorticoids was accepted as first-line therapy, systemic glucocorticoids remain the primary therapeutic option for pemphigus patients, successfully decreasing morbidity and mortality from pemphigus. However, novel therapeutic strategies are desirable due to the low efficacy in some subset of patients and the long-term severe adverse effects of traditional therapies. Recently, immunotherapy has proved to be encouraging for disease control or cure. Based on the current understanding of the immune mechanisms of pemphigus, we review the immune targets and corresponding agents applied in practice or under clinical trials. The goals of the novel treatments are to improve the quality of life of pemphigus patients by improving efficacy and safety, minimizing side effects, achieving fast disease control, or curing the disease.

## Introduction

Pemphigus is an autoimmune and organ-specific bullous disease, with flaccid blisters and superficial erosions on the skin and mucous membrane of the patients. Two primary types are pemphigus vulgaris (PV) and pemphigus foliaceus (PF), of which PV is more common than PF (1). Diagnosis is based on the intraepidermal blister and acantholysis by histology, IgG deposition between acanthocytes by direct immunofluorescence study (DIF), and positive serologic IgG by indirect immunofluorescence study (IIF) or anti-Desmoglein 3 or 1 (Dsg3 or 1) autoantibodies by ELISA (2). The current mainstay therapy for pemphigus is systemic glucocorticoids, as administered in most other autoimmune diseases (3, 4). However, prolonged application of glucocorticoids often leads to many adverse effects, such as Cushing's syndrome, infectious complications, dysregulation of the hypothalamic-pituitary-adrenal axis, hypertension, hyperglycemia and osteoporosis (4, 5). Consequently, topical treatment with strong glucocorticoids is often chosen in clinical practice to minimize the side effects caused by systemic application (6, 7). Additionally, other immunosuppressants such as azathioprine (AZA), methotrexate (MTX), cyclosporine A (CSA), mycophenolate mofetil, and cyclophosphamide (CTX) were also standard options for the treatment of pemphigus patients (8, 9). However, severe side effects such as infertility, increased risk of cancer, genitourinary complications, hypertension, lymphopenia, teratogenic effects, and infection have limited its use (10–15). In the past decade, a series of studies have helped better understand the immune mechanisms of pemphigus. A milestone work has been the successful application of CD20 monoclonal antibody (mAb) (16–18). Recently, a few novel targets for immunotherapy have been identified, and the biological and immunologic agents developed specifically against these targets could provide more effective therapies for pemphigus patients.

## Immune Mechanism Involved in Pemphigus Disease

Pemphigus is a life-threatening autoimmune bullous disease, and the patients have autoantibodies targeting the adhesion proteins (Dsg1 or 3) among keratinocytes, leading to acantholysis of skin and mucous membrane. The autoantibodies disrupt desmosomal Dsgs through steric hindrance, activation of transmembrane signaling, internalization, and intracellular degradation that down-regulates cell-cell adhesion (19–23). Current evidence has supported that autoreactive T cells, B cells, and the cytokines regulating their function are critical in developing autoimmunity and production of autoantibodies in pemphigus.

B cells have assumed a prominent position in producing pathogenic autoantibodies and contributing to antigen presentation and immune co-stimulation, suggesting that depleting B cells may be a practical approach for pemphigus therapy (24). Several novel therapeutic strategies targeting B cells have been in investigational or clinical trials for the treatment of pemphigus, and those included anti-CD20 antibodies and Bruton's tyrosine kinase inhibitors (BTKI) targeting B cell receptor signaling (25).

Several studies have indicated the importance of T cells in pemphigus (26, 27) and the role of Dsg3-specific CD4^+^ T cells has been elegantly demonstrated in an animal model by inducing a phenotype of interface dermatitis and PV (28), and defective regulatory T (Treg) cells may play a role in the onset of pemphigus by modulating the production of anti-Dsg3 autoantibodies (29). Tsunoda et al. have demonstrated that the interaction between autoreactive T cells and B cells was the key event for humoral autoimmunity targeting Dsg3 by transferring Dsg3-specific T cells or B cells into Dsg3^+/+^ Rag2^−/−^ mice (30). Therefore, the T and B immune axis involved in the pemphigus immune mechanisms may serve as primary therapeutic targets for patients with pemphigus.

In addition to the immune cells, cytokines, a group of low molecular weight proteins produced during immune responses, act as a signaling mediator that allows complex interactions between lymphocytes. By binding to specific receptors in the target cells, they initiate a cascade of intracellular signaling leading to the regulation of important biological functions, such as the growth, activation, differentiation, survival and death of the cells (31, 32). Numerous factors promoting B-cell differentiation, function and survival have been identified, including TNF-α, IL-1, IL-2, IL-4, IL-6, and IL-10 (33). B-Lymphocyte Stimulator (BLyS, also called B-cell Activating Factor, BAFF) and APRIL (A Proliferation-Inducing Ligand) are members of the TNF superfamily that play an essential role in B-cell survival and proliferation (34). Thus, targeting these cytokines to inhibit the proliferation and activation of B cells may represent a new approach to disease therapy.

## Therapeutics Targeting B Cells and B Cell Activation

### Rituximab

Rituximab is a monoclonal IgG1 antibody against CD20^+^ B cells (35). This antibody was studied in a prospective, multicenter, parallel-group, and open-label randomized trial and was granted a Breakthrough Therapy Designation by the US FDA for the initial treatment of PV. Subsequently, rituximab was accepted as a first-line therapeutic option when combined with short-term systemic corticosteroids (36–39). Additionally, high-dose rituximab was associated with a longer duration of complete clinical remission than low-dose rituximab (40). Long-term analysis of patients with pemphigus who received rituximab have shown that relapse was linked to the same anti-Dsg B cells observed during active disease, supporting that relapse resulted from the incomplete depletion of the autoreactive B cells clones (41). In addition to relapse, resistance to rituximab therapy could emerge during treatment, which could occur due to either genetic polymorphisms or the development of human anti-chimeric antibodies against the murine fragment of rituximab, preventing the drug from binding to B cells (42). Rituximab therapy also showed a risk of developing serious adverse events such as infection and hypogammaglobulinaemia (43). To improve the effectiveness and tolerability, new immunotherapy agents are currently under investigational trials.

### Next-Generation Anti-CD20 Monoclonal Antibodies

Anti-CD20 antibodies are diverse and could be categorized as type I and type II according to the cellular response upon binding. Type I mAbs localize CD20 into lipid rafts on the plasma membrane, leading to clustering of CD20 that enhances the recruitment and activation of complement (44, 45). In contrast, Type II mAbs exhibit stronger homotypic adhesion and more direct induction of cell death than type I mAbs, albeit with a minimal complement-dependent cytotoxic (CDC) response.

**Veltuzumab** is so far the only next-generation anti-CD20 mAb that has been reported in the treatment of refractory PV patients. This antibody is a type I, humanized anti-CD20 mAb with framework regions of epratuzumab, a humanized anti-CD22 antibody. It's significant advantage over rituximab is that it can be administered subcutaneously in low doses, making it more convenient to be applied on patients (46).

**Ofatumumab** is a type I, fully human, anti-CD20 monoclonal antibody, which targets an epitope of CD20 different from the rituximab binding site and has been proved to be safe and effective for the treatment of lymphoproliferative and other autoimmune disorders (47). A phase III randomized placebo-controlled trial of subcutaneous ofatumumab in pemphigus was recently terminated in 2018 (NCT01920477), and the results of this study remain to be reported (48).

Additionally, **ocrelizumab**, **obinutuzumab/GA-101**, **ocaratuzumab (AME-133v)**, and **PRO131921**, which are the third-generation anti-CD20 mAbs used for treating relapsing multiple sclerosis (49, 50) and chronic lymphocytic leukemia (CLL) with coexisting conditions (51, 52), representing promising therapeutic options for pemphigus in the future (38). In addition, the monoclonal antibody against CD19, **inebilizumab**, is considered an effective treatment for pemphigus patients who showed resistance to rituximab treatment due to the expression of CD19 on both B cells and plasmablasts (25, 53).

### Bruton Tyrosine Kinase Inhibitors

Bruton tyrosine kinase (BTK) is an enzyme that plays a vital role in the signaling transduction in most white blood cells other than T cells and plasma cells. BTK inhibitors (BTKI) are small molecules downregulating various B-cell activities, including cell proliferation, differentiation, maturation, and survival. Thus, BTKI are capable of suppressing the production of pemphigus autoantibodies (54). Among them, **PRN1008 (rilzabrutinib)** is a BTK inhibitor that was safe and well-tolerated following oral administration, and the report of a phase I study treated with PRN1008 demonstrated that PRN1008 could be effective on pemphigus. Moreover, PRN1008 has been granted Orphan Drug Designation by the United States FDA for PV therapy (55). Phase II trial of rilzabrutinib has been completed, and the result showed that rilzabrutinib alone or with low doses of corticosteroid was safe with rapid clinical activity in pemphigus vulgaris patients (56). Additionally, Jun Yamagami et al. investigated the efficacy and safety of tirabrutinib, another BTK inhibitor, in patients with refractory pemphigus in a multicenter, open-label, uncontrolled, single-arm phase II study. They reported that treatment with tirabrutinib enabled remission and reduced oral corticosteroids over time without significant safety concerns in patients with refractory pemphigus (57). Interestingly, another BTK inhibitor (PRN473) has been reported with a good response in canine pemphigus foliaceus (PF) (58).

### Target to T Cell and T-B Cell Interaction

The importance of T cells in orchestrating autoimmune reactions and efficient autoantibody production has been highlighted. **Daclizumab** and basiliximab (mAbs against CD25), have been developed as immunosuppressive drugs for patients after transplantation (59). It was used to successfully treat a PV patient who responded favorably to daclizumab in combination with prednisolone and azathioprine after a combination of conventional therapies failed (60). These data suggest that daclizumab and other anti-CD25 antibodies could provide an alternative treatment for recalcitrant pemphigus.

Additionally, **CD40/CD154** and **ICOS/ICOS-L** interaction, **altered peptide ligands (APLs)**, and **p38 mitogen-activated protein kinase (p38MAPK)** signaling are believed to play differential roles in activating adaptive immune responses (26, 61, 62) or blister formation in the pathogenesis of pemphigus (63), potentially providing new targets for the treatment of pemphigus.

### Cytokine Inhibitors

As the concentration of TNF-α in the local skin lesions is elevated (64, 65), inhibition of TNF-α by **infliximab** or **etanercept** could be a successful treatment for pemphigus vulgaris in a few studies (66, 67). However, disease relapse has been reported in PV patients co-administrated with prednisone and infliximab after prednisone was tapered (68).

A recent case report showed the effectiveness of **tocilizumab**, a humanized mAb inhibiting IL-6 in the treatment of a patient with refractory PF and Behcet's disease, by blocking the IL-6 receptor binding site and regulating the immune responses (69, 70).

IL-4 is a key cytokine that is supposed to play a critical role in pemphigus. **Dupilumab**, a fully human mAb directed against the IL-4Rα blocking IL-4 related to IL-13 signaling (71), could be a therapeutic option for pemphigus (72).

B-cell-activating factor (BAFF) is one of the TNF family members and an essential regulator of peripheral B-cell survival, maturation, antibody production, and class-switching (73, 74). A TNF receptor superfamily member 13C is expressed in most B cell subsets, promoting the survival of naive B cells and plasmablasts (75). The monoclonal antibody **VAY736** targeting this receptor may have a broad range of effects on B cell depletion and plasmablast survival, and the phase II clinical study is under clinical trials to examine the efficacy in treating pemphigus (NCT01930175) (42).

In addition, a proliferation-inducing ligand (APRIL), another TNF superfamily ligand, is also implicated in B-cell ontogeny (76) and may become another target for pemphigus therapy. However, further studies are necessary to clarify the exact role of APRIL in this skin condition.

## Other Inhibitors and mAbs

### FAS Ligand Inhibitor

An experimental study showed that soluble Fas ligand, which is upregulated and released from keratinocytes, was believed to play a critical role in blistering in the pemphigus pathogenesis (77). In accordance with this observation, a novel anti-soluble Fas ligand human monoclonal antibody (**PC111**) has been tested for pemphigus therapy due to its low potential for immunogenicity, favorable chemical and physical stability, and high binding affinity (78).

### Neonatal Fc Receptor Inhibitor

Owing to the role of neonatal Fc receptor (FcRn) in autoantibody production, FcRn could be a promising therapeutic target for treating IgG-mediated autoimmune disorders by preventing the persistent autoantigen presentation and consequently inhibiting long-term autoantibody production (79, 80). **SYNT001** (ALXN1830), a novel humanized IgG4 monoclonal antibody targeting FcRn at the immunoglobulin G (IgG) binding site, is considered another option for pemphigus therapy (81). More recently, the US Food and Drug Administration (FDA) has granted Orphan Drug Designation to SYNT001 to treat pemphigus in 2018 (38). Additionally, efgartigimod (ARGX-113) is an engineered Fc fragment derived from human IgG1 (82). A phase II, open-label study of efgartigimod in patients with pemphigus vulgaris and pemphigus foliaceus showed that efgartigimod induced early decrease of anti-desmoglein 1 and 3 autoantibodies in serum, representing a well-tolerated option of achieving early disease control and complete clinical remission of pemphigus while early corticosteroid tapering (83).

## Novel Cell Therapy

### Dsg-Specific B Cell Depletion by Chimeric Antigen Receptor Therapy

In order to eliminate the antigen-specific B cells that produce antibodies, Ellebreht et al. created a chimeric autoantibody receptor (CAAR), with the autoantigen Dsg3 as the CAAR extracellular domain, to engineer T cells to deplete the autoimmune memory B cells directly and Dsg3-specific short-lived plasma cells indirectly in PV patients (84). Dsg3-CAART therapy has been reported to lead to serological and histological improvements in experimental pemphigus mice without detectable off-target toxicity (85). However, the human study that assesses its efficacy and safety in humans is still needed. Nevertheless, the successful development of this strategy may lead to the generation of long-term memory CAAR-Tregs that could potentially cure the disease.

### Polyclonal Regulatory T Cells (PolyTregs) Therapy

The immune system is a complex network and a large amount of evidence has verified the role of Tregs in regulating the immune system and preventing autoimmune diseases (86). There has been a clinical trial of Treg adoptive therapy treating graft vs. host diseases (GVHD) with expanded allogeneic Tregs (87), and another study demonstrated that the administration of autologous Tregs was safe and the disease activity of patients with insulin-dependent diabetes decreased (88). Additionally, a non-randomized, open-label, phase I clinical trial is under investigation (NCT03239470) to evaluate the effects of autologous expanded Tregs on the PV (48).

### Autologous Hematopoietic Stem Cell Transplantation

The efficiency of Autologous hematopoietic stem cell transplantation was reported in both PV and PF patients, together with a high risk of serious adverse events (89–92). Therefore, the effectiveness and safety of this strategy need to be further evaluated and verified by a long-term, large cohort study.

## Conclusion

**Figure 1:**
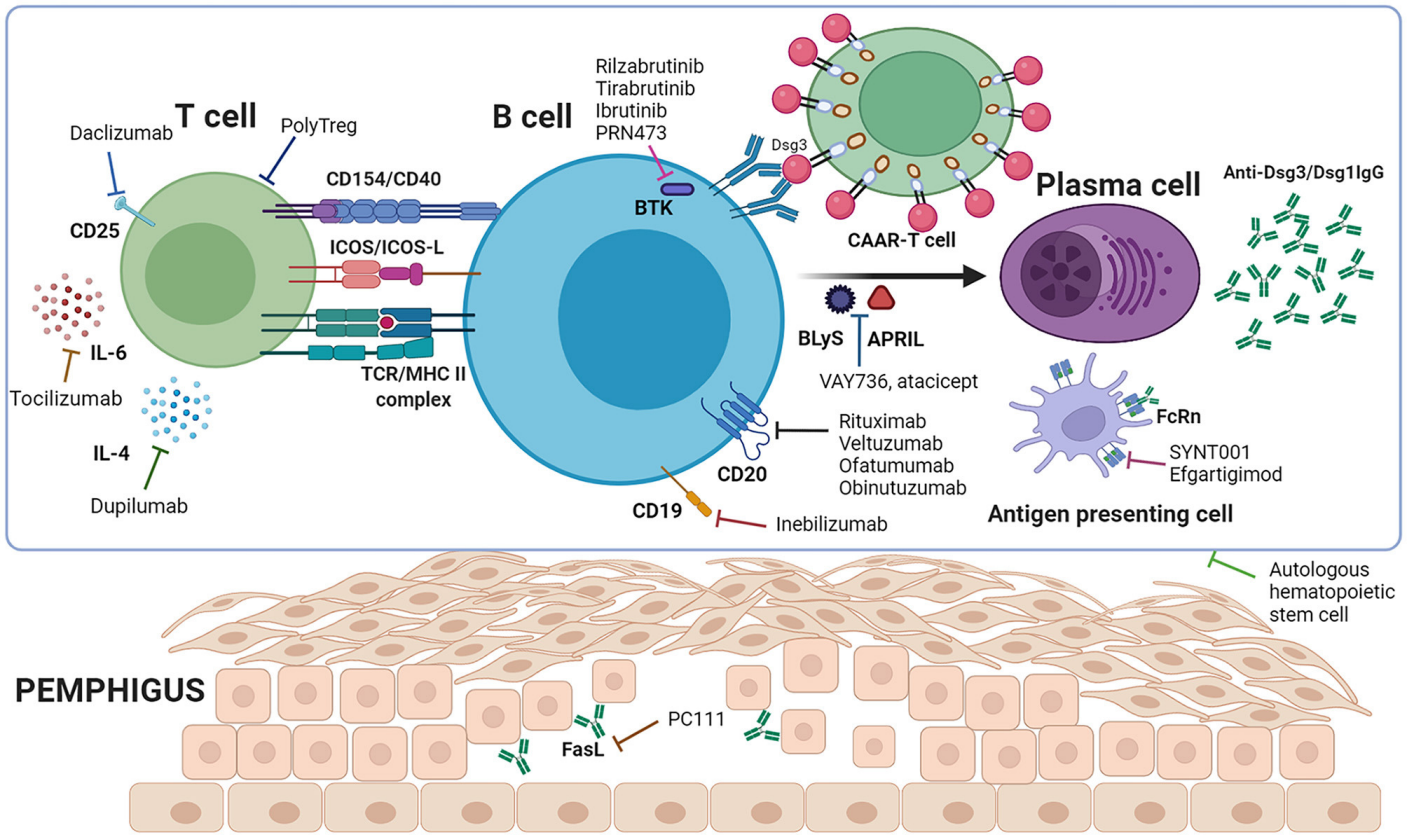
Immune mechanism of pemphigus and targeted therapeutic agents. T cells interact with B cells to provide co-stimulatory signals through CD154/CD40, ICOS/ICOS-L etc., leading to B cell activation, proliferation, and differentiation to plasma cells, and secretion of anti-Dsg3/Dsg1 autoantibodies. Binding of the antibodies to the target antigen among acanthocytes leads to the separation of keratinocytes and intraepidermal blister formation. Rituximab, veltuzumab, ofatumumab, obinutuzumab and inebilizumab deplete autoreactive B cells to prevent their differentiation to plasma cells. PolyTregs, daclizumab, tocilizumab and dupilumab act on T cells, while while rilzabrutinib, tirabrutinib, ibrutinib, PRN473, VAY736 and atacicept target B cells, resulting in less activation of autoreactive B cells. CAAR-T cells work to eliminate Dsg3-specific B cells. SYNT001 and efgartigimod saturate FcRn to shorten the half-life of pathogenic IgG autoantibodies. Autologous hematopoietic stem cells function by eliminating autoreactive lymphocytes and re-establishing the immune system (Created with BioRender.com).

**Figure 2:**
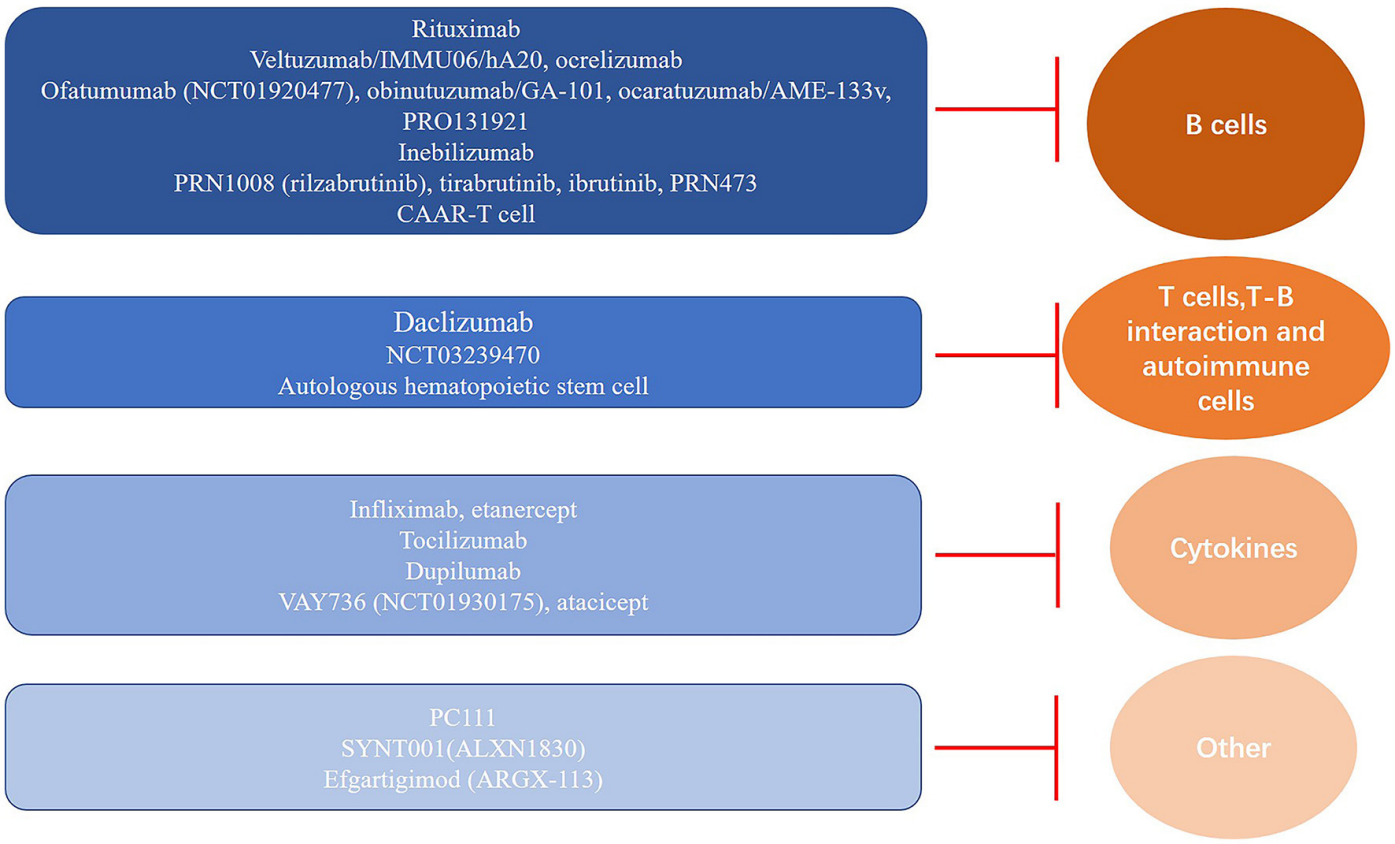
Immune pathomechanism-based targets and classification of therapeutic agents for pemphigus. Overview of the current and candidate agents for immunotherapy and their corresponding targets, including B cells, T cells, and cytokines, which are essential for pathogenic autoantibody production and secretion in pemphigus.

In summary, we have shown the current pemphigus immunotherapies, including biological agents and cell therapy strategies (Table 1) investigated for the clinical treatment of pemphigus and undergoing clinical trials (Figure 1). These therapies are primarily based on the current understanding of pemphigus disease pathology. Pemphigus disease is mainly mediated by circulating autoantibodies against Dsgs. These antibodies are expressed and secreted by Dsg3 autoreactive B cells that are activated presumably by the autoreactive T cells, in which cytokines could also play an essential role in pemphigus disease pathophysiology (Figure 2). The autoantibodies disrupt desmosomal Dsgs by the assembly and disassembly pathways (93). A few review papers have recently been published and described the potential therapies for pemphigus targeting these pathways (53, 94–97). The current review focuses on the immune mechanism-based therapies to target the Dsg3-specific B cells, T cells, and relevant cytokines. In future research, more efforts should be paid to minimize the adverse effects of conventional therapies and reduce the relapse frequency. The ultimate goal is to achieve rapid disease control, complete disease remission, and disease cure. With the accumulation of the knowledge of pemphigus pathogenesis, novel targets could be identified, and more therapeutic agents with improved efficacy will be developed and applied for PV management in clinical practice.

**Table 1:** Biological agents of immunotherapy and their status of clinical trial for pemphigus.

**Target**	**Category**	**Approved**	**Under trial**	**Candidates**
B cell	CD20 mAb (First generation)	Rituximab		
	CD20 mAb (Second generation)			Veltuzumab/IMMU06/hA20 Ocrelizumab
	CD20 mAb (Third generation)		Ofatumumab (NCT01920477)	Obinutuzumab/GA-101 Ocaratuzumab /AME-133v PRO131921
	CD19 mAb			Inebilizumab
	BTK inhibitor	PRN1008 Rilzabrutinib	Tirabrutinib (Finished phase II trial)	Ibrutinib, PRN473
Dsg3-specific B cells			CAAR-T cell	
T cell and T-B interaction	CD25			Daclizumab
	PolyTregs		NCT03239470	
Autoimmune cells				Autologous hematopoietic stem cell
Cytokines	TNF-α			Infliximab Etanercept
	IL-6			Tocilizumab
	IL-4			Dupilumab
	BAFF (BLys and APRIL)		VAY736 (NCT01930175)	Atacicept
Other	Fas ligand			PC111
	FcRn	SYNT001 (ALXN1830)	Efgartigimod (ARGX-113, phase II trial finished)	

## Author Contributions

HY organized the database and wrote the first draft of the manuscript. HY and XM contributed to the manuscript revision. All the authors contributed to the conception and design of the review, read, and approved the submitted version.

## Funding

This work was supported by the National Natural Science Foundation of China (Nos. 81803128, 81730085, and 81974475) and the Shanghai Sailing Program (No. 18YF1414200).

## Conflict of Interest

The authors declare that the research was conducted in the absence of any commercial or financial relationships that could be construed as a potential conflict of interest.

## Publisher's Note

All claims expressed in this article are solely those of the authors and do not necessarily represent those of their affiliated organizations, or those of the publisher, the editors and the reviewers. Any product that may be evaluated in this article, or claim that may be made by its manufacturer, is not guaranteed or endorsed by the publisher.
